# Experimental exposure to climate change scenarios imposed alterations on the morphological traits of sessile and low-motility marine invertebrates

**DOI:** 10.3897/BDJ.14.e186719

**Published:** 2026-03-10

**Authors:** Eva Chatzinikolaou, Kleoniki Keklikoglou, Emmanouela Vernadou, Thanos Dailianis

**Affiliations:** 1 Hellenic Centre for Marine Research (HCMR), Institute of Marine Biology, Biotechnology and Aquaculture (IMBBC), Heraklion, Crete, Greece Hellenic Centre for Marine Research (HCMR), Institute of Marine Biology, Biotechnology and Aquaculture (IMBBC) Heraklion, Crete Greece

**Keywords:** micro-computed tomography (micro-CT), *Hexaplex trunculus*, *Chondrilla nucula*, gastropods, sponge, warming, ocean acidification, morphology

## Abstract

**Background:**

Over the past 50 years, the oceans have absorbed over 90% of global warming heat, leading to warming, acidification and declining oxygen levels that are disrupting marine ecosystems and altering species distributions and productivity. The vulnerability of marine organisms to these changes depends on their biological traits, habitat conditions and adaptive capacity, influencing their growth, behaviour and overall population health. Micro-computed tomography (micro-CT) has been previously used for studying the morphological traits of marine invertebrates, which provide important insights into species functionality and responses to climate change and ocean acidification. Micro-CT enables non-destructive, high-resolution 3D analysis of internal and external structures, allowing precise measurement of traits such as density, porosity and morphology that are valuable for climate change research.

**New information:**

The present manuscript describes micro-CT imaging datasets generated to investigate the effects of climate change on the morphological structure of two benthic marine invertebrates: the low-motility gastropod *Hexaplex trunculus* (Linnaeus, 1758) and the sessile sponge *Chondrilla nucula* Schmidt, 1862. Both species are considered particularly vulnerable to environmental stressors. To date, no study has investigated the effects of ocean warming and acidification on sponges using micro-CT technology. Using a common garden experimental design, individuals from geographically distinct populations exposed to different natural environmental regimes were subjected to combined warming and acidification scenarios to assess their morphological responses and adaptive capacity.

## Introduction

The oceans have absorbed most of the planet’s warming over the past 50 years, thus acting as a heat reservoir with more than 90% of the heat gain occurring within their waters (Venegas et al. 2023). Climate change is driving significant changes in the ocean, including rising sea temperatures, ocean acidification and declining oxygen levels. These shifts are disrupting marine ecosystems, altering species abundance and distributions and affecting the productivity and resilience of ocean life (Venegas et al. 2023). At the organismal level, ocean warming and acidification can affect growth, development, reproduction, metabolism and behaviour of marine species, thus impacting their population balance and overall health (Christou et al. 2025). The vulnerability of an organism depends on its inherent biological or ecological traits — specifically its sensitivity and adaptive capacity — that determine how well it can withstand and respond to changing environmental conditions (Fortini and Schubert 2017). Organisms found in habitats with a naturally occurring narrow temperature range may be more sensitive to ocean warming, while calcifying ones might be highly affected by ocean acidification (Pacifici et al. 2015). Species-specific behaviour, interactions and adaptive responses also play a key role in determining their vulnerability to a changing ocean (Pacifici et al. 2015).

Morphological traits can offer valuable insights into species functionality, especially when they can reveal internal structures and micro-structures at an intact state. Specific morphological features, such as the calcified structures (e.g. shell), have also been used as responsed traits to climate change and ocean acidification (Byrne and Fitzer 2019, Gray et al. 2022). Micro-computed tomography (micro-CT) is a high-resolution, X-ray–based three-dimensional imaging technique that has been employed in several studies to investigate the effects of climate change and ocean acidification on marine organisms (Chatzinikolaou et al. 2016,Di Santo 2019, Fordyce et al. 2020, Leung et al. 2025, Chatzinikolaou et al. 2021a, Chatzinikolaou et al. 2016Leung et al. 2025). This technique enables non-destructive examination of both external and internal structures of a specimen, resulting in its complete virtual representation in three dimensions. The use of the most appropriate parameters, such as voltage, scanning medium, filters, staining agents, exposure time, magnification etc., are important for achieving an optimum imaging result (Chatzinikolaou and Keklikoglou 2021). The ability to perform three-dimensional analyses for the calculation of parameters, such as density, porosity, structural thickness and geometric morphometric metrics (Keklikoglou et al. 2019), which may serve as useful traits in climate change research, represents an additional advantage of this technology for climate change studies.

Micro-CT has previously been applied to the study of morphological properties of marine invertebrates, particularly gastropods (Chatzinikolaou et al. 2016, Rühl et al. 2017, Barclay et al. 2020, Chatzinikolaou et al. 2021a). Shell density and thickness have been used as morphological traits in ocean acidification studies, as alterations in these parameters have been observed in several investigations. For example, Chatzinikolaou et al. (2021a) quantified shell morphological properties in the gastropods *Nassarius nitidus* and *Columbella rustica*, showing that both species developed less dense and thinner shells under low-pH conditions. Building on this body of work, the present manuscript extends the application of micro-CT to different taxa by investigating the morphological properties of a previously unexamined gastropod species and a sponge. Particularly for sponges, to our knowledge, no study has yet investigated the effects of climate change and ocean acidification using micro-CT technology.

### Aim of the project

The aim of the project was to study the impact of climate change on the morphological structures of two species, the low-motility marine gastropod *Hexaplex trunculus* (Linnaeus, 1758) (Mollusca, Gastropoda) and the sessile sponge *Chondrilla nucula* Schmidt, 1862 (Porifera, Demospongiae). Sessile and low-motility marine invertebrates are particularly vulnerable to environmental pressures due to their limited ability to avoid unfavourable conditions. The proposed approach involved experimental exposure of the targeted organisms to climate change scenarios combining elevated temperature and reduced pH gradients, thereby realistically simulating the synergistic impact of naturally occurring stressors, more specifically ocean warming and acidification. Individuals collected from a North Aegean and a South Aegean population in Greece, were used in a common garden experiment in order to assess the responses of these organisms to thermal and oxidative stress, with the additional aim of investigating intraspecific variation in these responses and thereby uncovering potential adaptive diversity amongst geographically distinct populations.

## Project description

### Title

Multi-level Approaches to assess Climate Change Impact to Marine Organisms (MACCIMO)

### Personnel

Dr Thanos Dailianis (scientific responsible, experimental design), Dr Eva Chatzinikolaou (experimental design, statistical analysis, WP2 leader), Dr Kleoniki Keklikoglou (sample scanning, image analysis, WP3 leader), Emanouela Vernadou (sample scanning, image analysis)

### Study area description

Crete - South Aegean (35.3357° N, 25.2815° S, up to 5 m depth) and Chalkidiki - North Aegean (39.9315° N, 23.7348° S, up to 5 m depth), Mediterranean Sea, Greece

### Design description

Three climate change scenarios were simulated during the experiments: 1) the Control scenario in which the ambient temperature was estimated as the average of the maximum summer temperatures (27°C) between the two populations (north and south Greece). The pH was ambient (~ 8.1); 2) the South Aegean Climate Change (SACC) scenario ("extreme") in which temperature was estimated as the maximum recorded in South Aegean (Crete) during summer (27°C) increased by 4°C. The pH was decreased by 0.3 units (~ 7.8); and 3) the North Aegean Climate Change (NACC) scenario ("mild") in which temperature was estimated as the maximum recorded in North Aegean (Chalkidiki) during summer (26°C) increased by 4°C. The pH was decreased by 0.3 units (~ 7.8).

The selected Climate Change scenarios (i.e. final temperature increase by 4°C and final pH decrease by 0.3 units) were based on the "high GHG emissions" RCP 8.5 scenario of IPCC as this is described in the Climate Change 2023: Synthesis Report (Calvin et al. 2023).

### Funding

This work was funded by the Hellenic Foundation for Research and Innovation (HFRI) under the “2nd Call for HFRI Research Projects to support Faculty Members & Researchers”, Project Number 3280.

## Sampling methods

### Sampling description

**Selection of species**

The sponge *C. nucula* and the gastropod *H. trunculus* were selected as the experimental organisms in this study. *C. nucula* is a photophilic sponge with a modular growth form, consisting of clonal globular body masses, often covering extensive patches on well-lit hard substrates (Milanese et al. 2003). It is a widespread species found across a variety of habitats usually in coastal shallow waters from the sea surface down to 30 m depth (Strano et al. 2020), but also in deeper waters (Stamouli et al. 2023). As with most poriferan taxa, *C. nucula* is a filter-feeder, actively pumping water through its body to remove particulate matter, thus playing an important role in maintaining water quality through nutrient recycling in marine ecosystems (Milanese et al. 2003). It is a very efficient competitor for space, a characteristic that makes it the dominant species in some benthic habitats (Strano et al. 2020). *C. nucula* is also of great commercial interest due to its bioactive compounds (Chelossi et al. 2007), making it a candidate for biotechnological applications.

*H. trunculus* is a cosmopolitan species found in the intertidal and subtidal zone and it is well adapted and resistant to changing environmental conditions (Lahbib et al. 2010). It reproduces by egg capsules from which young individuals hatch directly (direct development) without the intervention of a planktonic larval stage (Lahbib et al. 2012). *H. trunculus* feeds on live and/or dead organisms (e.g. bivalves, gastropods) (Peharda and Morton 2005) and is easily maintained in laboratory tanks. It is an edible species with commercial value in many countries (e.g. Italy, Tunisia, Portugal and Spain) (Vasconcelos et al. 2006,Lahbib et al. 2012). It is also an important ecological indicator for the pollution of the marine environment by organotin compounds (e.g. TBT) (Abidli et al. 2012).

**Experimental set-up**

The experimental set-up, used to adjust the treatment tanks to the desired levels of temperature and pH, as well as the protocols for the maintenance of organisms during the 3 months of the experiment, have been described in detail in the first manuscript of the Special Issue series "Multi-level assessment of climate change impacts in benthic marine invertebrates: insights from the MACCIMO project" which is published by Chatzinikolaou et al. (2026).

**Collection of samples**

A group of five individuals from each population (northern and southern) per treatment were randomly sampled for scanning in the micro-CT following the end of the experiment after 3 months. Specimens were anaesthetised using a rising concentration of MgCl_2_ starting at 1.5% and gradually reaching 3.5% according to the European Directive 2010/63 EU on the protection of animals used for scientific purposes. The samples were stored at 20 ͦC until micro-CT scanning was performed.

**Scanning equipment**

Micro-CT scans were performed with a SkyScan 1172 micro-tomograph (Bruker, Kontich, Belgium) at the Hellenic Center for Marine Research (HCMR) - Institute of Marine Biology, Biotechnology and Aquaculture (IMBBC). This scanner uses a tungsten source and is equipped with an 11MP CCD camera (4000 x 2672 pixels), which can reach a maximal resolution of < 0.8 μm/pixel.

**Scanning protocols for *Chondrilla nucula***

Sample preparation: *C. nucula* specimens were fixed in 4% formalin for 3 days and subsequently washed with distilled water to remove the remaining formalin. Α fragment (~ 30 mm^3^) from each specimen was cut and used for micro-CT scanning in order to maximise the resolution of the images. The sponge fragments were dehydrated in a graded series of ethanol solutions (20%, 50%, 70% and up to 96%) and remained for 24 hours in each solution. Hexamethlydisilizane (HMDS) was selected as a contrast agent in order to increase the contrast of soft tissues. HMDS is a drying agent as it has the ability to remove water from the cells and the surrounding area resulting in greater contrast of tissues (Faulwetter et al. 2013, Paterson et al. 2014). The drying procedure of Paterson et al. (2014) was used for *C. nucula* specimens. Specifically, specimens stored in 96% ethanol were submerged into 1:1 solution of 96% ethanol and HMDS for 24 hours. Subsequently, specimens were transferred to 100% HMDS for 24 hours. Finally, the specimens were air dried overnight under a laboratory hood.

Scanning procedure: *C. nucula* specimens were placed and stabilised inside a pipette tip which serves as a suitable sample holder due to the low X-ray absorption of polypropylene. Specimens were scanned in air at a voltage of 60 kV and a current of 167 μA without any filters. Images were acquired at a pixel size of 2.97 μm with a camera binning of 1×1. Exposure time was 315 ms and scans were performed for a full rotation of 360°, a rotation step of 0.21° and a frame averaging of 3. Scanning duration was around 2 hours and 10 minutes for each specimen. Projection images were reconstructed into cross-sections using SkyScan’s NRecon software (Bruker, Kontich, Belgium) which employs a modified Feldkamp’s back-projection algorithm. Scans were reconstructed in a range of attenuation coefficients of 0-0.26, with a beam-hardening correction of 59%, smoothing of 2 and ring artefact correction of 20. The reconstructed images were stored as 16-bit TIFF images.

3D analysis: 3D analysis was performed in the reconstructed images using the CT Analyser software (CTAn, Bruker, Kontich, Belgium). The relative density of each *C. nucula* specimen was calculated using the mean grey scale values applied by the binary threshold module of the CTAn as a proxy. A range of 20-255 of the grey-scale histogram was used, which allowed comparable measurements of the sponges tissue relative density.

**Figure 1.**
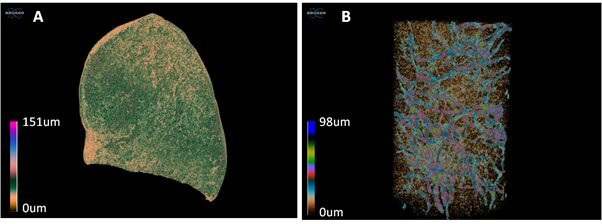
Colour-coded images of *Chondrilla nucula* specimens showing: A) the structure thickness of the soft tissue and B) the pore network. Each colour represents a different thickness and pore size class, respectively.

Volume renderings of each specimen were created using the CTVox software (Bruker, Kontich, Belgium) in order to display the 3D structure thickness, along with the pores network of the sponges, corresponding to the canals of the aquiferous system (Fig. 1). The total, closed and open porosity (%) for all specimens were calculated using the custom processing plugin of CTAn. Total porosity was calculated as the percentage (%) of the total volume of pores in relation to the total volume of each specimen. Open porosity refers to all the pores or spaces which are connected to the outer surface of the specimen, while closed porosity refers only to the enclosed pores.

Structure thickness refers to the thichkness of the specimen tissue, whereas structure separation refers to the thickness of the spaces (pores) and is an estimate of the pores size (diameter). Both structure parameters are calculated as the average of the diameters of the largest spheres which can be fitted into each point of the specimen structure ("sphere-fitting" method) (Hildebrand and Rüegsegger 1997).

**Scanning protocols for *Hexaplex trunculus***

Sample preparation: *H. trunculus* specimens were fixed in 4% formalin for 3 days and subsequently washed with distilled water in order to remove the remaining formalin. Specimens were dehydrated in a graded series of ethanol solutions (20%, 50%, 70% and up to 96%) and remained 24 hours in each solution. No contrast enhancement method was needed as the shell is a dense material with high X-ray absorption.

Scanning procedure: The scanning protocol used is in accordance with the methodology described by Chatzinikolaou and Keklikoglou (2021). Specimens were placed and stabilised inside a custom-made styrofoam holder which is a suitable sample holder due to its low X-ray absorption. Scans were performed at a voltage of 100 kV and a current of 100 μA using a combined aluminium and copper filter. Images were acquired at a pixel size of 13.79 μm with a camera binning of 2×2. Exposure time was 2480 ms and scans were performed for a full rotation of 180°, a rotation step of 0.6° and without frame averaging. Scanning duration per specimen was about 6 hours. Projection images were reconstructed into cross-sections using SkyScan’s NRecon software (Bruker, Kontich, Belgium) which employs a modified Feldkamp’s back-projection algorithm. Scans were reconstructed in a range of attenuation coefficients of 0-0.15, with a beam-hardening correction of 59%, smoothing of 2 and ring artefact correction of 20. The reconstructed images were stored as 16-bit TIFF images. Volume renderings of the 3D specimen were created using the CTVox software (Bruker, Kontich, Belgium).

3D analysis: 3D analysis was performed in the reconstructed images using the CT Analyser software (CTAn, Bruker, Kontich, Belgium). The relative density was calculated as the mean grey scale values of the shell in a range of the grayscale histogram of 30-255. Calculation of the mean grey-scale values of the total shell using the binary threshold module of CTAn allowed comparable measurements of the relative density of the shell in *H. trunculus*. Relative grey-scale density was used as a proxy for estimating "micro-density" (i.e. density of the shell material including CaCO_3_ and intraskeletal organic matrix, excluding porosity) (Chatzinikolaou et al. 2021b).

**Figure 2.**
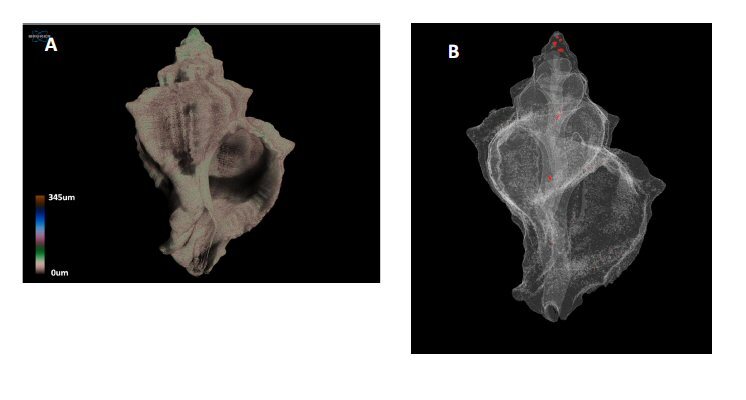
3D images produced following volume rendering in *Hexaplex trunculus* specimens for the visual presentation of: A) colour‐coded structure thickness, where each colour represents a different thickness class and B) the 3D model of the closed pores (indicated in red).

Using the custom processing plug-in of CTAn, the 3D structure thickness and the closed porosity (% of enclosed pores) of the shells were measured. Volume renderings of each specimen were created using the CTVox software (Bruker, Kontich, Belgium) in order to display the 3D structure thickness (Fig. 2A). The closed pores of the shells were visualised using the CTVol software (Bruker, Kontich, Belgium) (Fig. 2B).

The parameters of the scanning, reconstruction and 3D analysis procedure remained the same for all specimens of the same species in order to obtain comparable results between the different treatments.

## Geographic coverage

### Description

South Aegean - Crete (35.3357/25.2815)

North Aegean - Chalkidiki (39.9315/23.7348)

### Coordinates

35.3357 and 39.9315 Latitude; 23.7348 and 25.2815 Longitude.

## Taxonomic coverage

### Description

Phylum: Mollusca, Class: Gastropoda, Order: Neogastropoda, Family: Muricidae, Genus: Hexaplex, Species: *Hexaplex trunculus* (Linnaeus, 1758).

Phylum: Porifera, Class: Demospongiae, Order: Chondrillida, Family: Chondrillidae, Genus: Chondrilla, Species: *Chondrilla nucula* Schmidt, 1862.

### Taxa included

**Table:** 

Rank	Scientific Name	Common Name
species	*Hexaplex trunculus*	banded dye-murex
species	*Chondrilla nucula*	chicken-liver sponge

## Usage licence

### Usage licence

Other

### IP rights notes

The publisher and rights holder of this work is Hellenic Center for Marine Research. This work is licensed under a Creative Commons Attribution (CC-BY 4.0) License.

## Data resources

### Data package title

Micro-Computed Tomography (Micro-CT) Morphological Data of *Chondrilla nucula* and *Hexaplex trunculus* Subjected to Experimental Climate Change Conditions

### Resource link

https://doi.org/10.25607/vjxlve

### Alternative identifiers

https://ipt.medobis.eu/resource?r=micro-ct_morphological_data_chondrilla_nucula-hexaplex_trunculus GBIF UUID: 5dfb1134-a48d-4f45-bcbf-cb718b03e6e5

### Number of data sets

1

### Data set 1.

#### Data set name

Micro-Computed Tomography (Micro-CT) Morphological Data of *Chondrilla nucula* and *Hexaplex trunculus* Subjected to Experimental Climate Change Conditions.

#### Data format

Darwin Core Archive (DwC-A)

#### Download URL

http://ipt.medobis.eu/resource?r=micro-ct_morphological_data_chondrilla_nucula-hexaplex_trunculus

#### Data format version

Version 2.1

#### Description

This dataset includes 3D morphological data for two invertebrate species, the sponge *Chondrilla nucula* (Porifera, Demospongiae) and the gastropod *Hexaplex trunculus* (Mollusca, Gastropoda), which were produced using a micro-computed tomograph (micro-CT). More specifically, the morphological characters estimated for each specimen of the sponge *C. nucula* were Density (grey-scale values), Structure separation (um), Structure thickness (um), Closed porosity (%), Open porosity (%) and Total Porosity (%). The morphological characters estimated for each specimen of the gastropod *H. trunculus* were Density (grey-scale values), Structure thickness (um) and Closed porosity (%). Specimens of both species were subjected to experimental conditions simulating the climate change scenarios detailed above.

The dataset is available via the MedOBIS (Mediterranean node of Ocean Biodiversity Information System) Integrated Publishing Toolkit (IPT) which has been established through the LifeWatchGreece Research Infrastructure and is hosted in the Institute of Marine Biology, Biotechnology and Aquaculture (IMBBC) of the Hellenic Centre for Marine Research (HCMR). The data are also harvested by and made available through the Ocean Biodiversity Information System (OBIS) and through the Global Biodiversity Information Facility (GBIF). The dataset is available as a DarwinCore Archive and all fields are mapped according to DarwinCore terms.

The current publication refers to the "extended measurement or fact" source file (txt file) that is associated with the particular dataset. Additional details about the sampling events and the samples can be found in the "event" and "occurrence" source files, respectively (txt files), associated with the same dataset. This publication refers to the most recent version of the dataset available through the IPT server or MedOBIS. Future changes to the dataset due to quality control activities might change its content or structure.

**Data set 1.** 

Column label	Column description
id	A unique identifier for the record within the dataset or collection, auto-incrementing number automatically added by the system (same with eventID).
measurementID	An identifier for the dwc:MeasurementOrFact (information pertaining to measurements, facts, characteristics or assertions).
measurementType	The nature of the measurement, fact, characteristic or assertion. For the present study, the measurement types were Density (grey-scale), Structure separation, Structure thickness, Closed porosity, Open porosity and Total porosity.
measurementValue	The value of the measurement, fact, characteristic or assertion.
measurementAccuracy	The description of the potential error associated with the dwc:measurementValue.
measurementUnit	The units associated with the dwc:measurementValue.
measurementRemarks	Comments or notes accompanying the dwc:MeasurementOrFact.
institutionCode	The name (or acronym) in use by the institution having custody of the object(s) or information referred to in the record.
datasetName	The name identifying the dataset from which the record was derived.
eventID	An identifier for the set of information associated with a dwc:Event (something that occurs at a place and time).
parentEventID	An identifier for the broader dwc:Event that groups this and potentially other dwc:Events.
eventType	The nature of the dwc:Event.
fieldNumber	An identifier given to the dwc:Event in the field. Often serves as a link between field notes and the dwc:Event.
eventDate	The date-time or interval during which a dwc:Event occurred.
year	The four-digit year in which the dwc:Event occurred, according to the Common Era Calendar.
month	The integer month in which the dwc:Event occurred.
day	The integer day of the month on which the dwc:Event occurred.
samplingProtocol	The names of, references to or descriptions of the methods or protocols used during a dwc:Event.
eventRemarks	Comments or notes about the dwc:Event.
locationID	An identifier for the set of dcterms:Location information. May be a global unique identifier or an identifier specific to the dataset.
country	The name of the country or major administrative unit in which the dcterms:Location occurs.
countryCode	The standard code for the country in which the dcterms:Location occurs.
locality	The specific description of the place.
verbatimLocality	The original textual description of the place.
minimumDepthInMetres	The lesser depth of a range of depth below the local surface, in metres.
maximumDepthInMetres	The greater depth of a range of depth below the local surface, in metres.
decimalLatitude	The geographic latitude (in decimal degrees, using the spatial reference system given in dwc:geodeticDatum) of the geographic centre of a dcterms:Location.
decimalLongitude	The geographic longitude (in decimal degrees, using the spatial reference system given in dwc:geodeticDatum) of the geographic centre of a dcterms:Location.
geodeticDatum	The ellipsoid, geodetic datum or spatial reference system (SRS) upon which the geographic coordinates given in dwc:decimalLatitude and dwc:decimalLongitude are based.
coordinateUncertaintyInMetres	The horizontal distance (in metres) from the given dwc:decimalLatitude and dwc:decimalLongitude describing the smallest circle containing the whole of the dcterms:Location.
georeferenceProtocol	A description or reference to the methods used to determine the spatial footprint, coordinates and uncertainties.
basisOfRecord	The specific nature of the data record.
OccurrenceID	An identifier for the dwc:Occurrence (as opposed to a particular digital record of the dwc:Occurrence).
OccurrenceStatus	A statement about the presence or absence of a dwc:Taxon at a dcterms:Location.
IdentifiedBy	A list (concatenated and separated) of names of people, groups or organisations who assigned the dwc:Taxon to the subject.
IdentificationVerificationStatus	A categorical indicator of the extent to which the taxonomic identification has been verified to be correct.
ScientificNameID	An identifier for the nomenclatural (not taxonomic) details of a scientific name.
ScientificName	The full scientific name, with authorship and date information if known.
ScientificNameAuthorship	The authorship information for the dwc:scientificName formatted according to the conventions of the applicable dwc:nomenclaturalCode.

## Additional information

### Results - data analysis

The average values of the morphological parameters estimated for specimens of the sponge *C. nucula* and the gastropod *H. trunculus* from the two populations (north and south) and the three experimental treatments, namely Control (current conditions), South Aegean Climate Change (SACC) and North Aegean Climate Change (NACC), are presented in Table 1.

**Figure 3.**
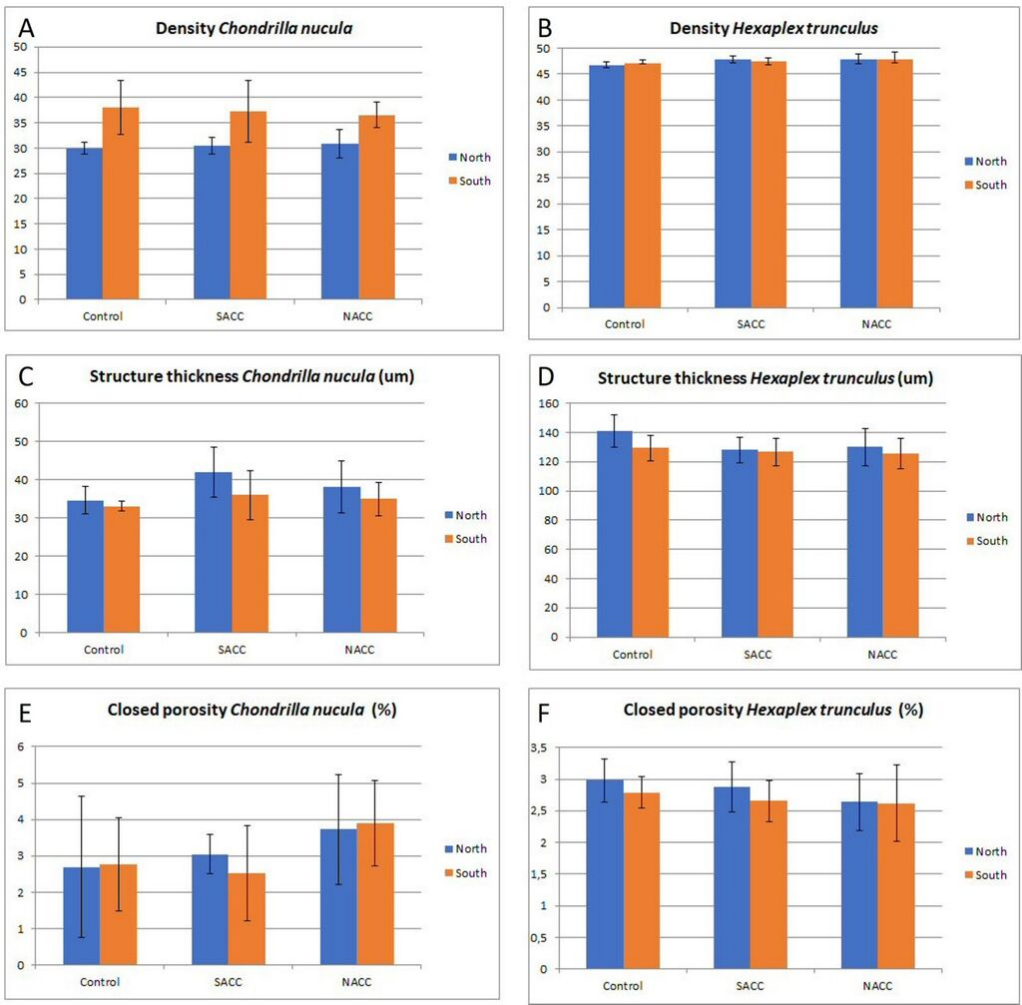
Graphical representation of the average values for density (grey-scale) (A and B), structure thickness (um) (C and D) and closed porosity (E and F) for *Chondrilla nucula* (left) and *Hexaplex trunculus* (right), from the two populations (North and South) and three treatments (Control, South Aegean Climate Change - SACC, North Aegean Climate Change - NACC). Error bars represent standard deviation.

Sponges (Porifera) are characterised by an extensive internal circulatory system, comprising of a network of choanoderm, pores and chambers (the aquiferous system), which is essential for their physiological functioning and development. Accordingly, for *C. nucula* specimens, total porosity, closed and open porosity, as well as structure thichkness and structure separation, were quantified. In contrast, for the more concrete shells of *H. trunculus*, the estimation of structure thickness and closed porosity were sufficient. Morphological parameters estimated for both species are presented in Fig. 3.

Shell density of *H. trunculus* is similar between individuals maintained in the two Climate Change treatments (SACC and NSCC), whereas the control specimens had a slightly lower average shell density (Fig. 3B). No profound differences were observed in density between specimens originating from the two different populations when compared for each treatment separately. Structure thickness was higher in the control treatment in comparison to the NACC and SACC treatments, especially for the *H. trunculus* originating from the north population (Fig. 3D). Similarly, *H. trunculus* that were maintained in the control had a higher closed porosity than the ones under NACC and SACC treatments (Fig. 3F). In addition, specimens from the south population had a lower closed porosity and a lower structure thickness in comparison to individuals coming from the northern population when compared for each treatment separately.

Density of *C. nucula* was similar in all treatments, but differences were evident between the two populations. In all cases, individuals from the south population had higher tissue density (Fig. 3). Specimens from the control treatment had lower closed porosity and lower structure thickness in comparison to both climate change treatments (NACC and SACC). More specifically, closed porosity was higher in specimens of *C. nucula* maintained under the less extreme climate change scenario (NACC). Structure thickness was higher for specimens maintained under the more extreme climate change scenario (SACC), especially for individuals originating from the north population.

### Conclusions

Three-dimensional analysis of the reconstructed micro-CT images enabled the estimation of morphological parameters — including density, porosity (closed, open and total), structure thickness and structure separation — in specimens of the sponge *C. nucula* and the gastropod *H. trunculus* that were maintained under the experimental treatments for a total period of 3 months. The effects of climate change scenarios combining elevated temperature and reduced pH were compared between the northern and southern populations of both species. A common-garden experimental design was used in order to realistically simulate the synergistic impact of co-occurring stressors and to reveal potential existing local adaptations.

The results of the present study indicated that exposure of the gastropod *H. trunculus* to higher temperatures and lower pH led to increased shell density, accompanied by reduced porosity and shell thickness. In contrast, specimens of the sponge *C. nucula* exhibited increased tissue porosity and thickness under the same experimental stressors. In addition, population-specific responses were observed in both selected species, in relation to geographical origin. Specifically, the southern population of *H. trunculus* showed relatively lower shell thickness and porosity, whereas the southern population of *C. nucula* displayed higher tissue density and again lower thickness. As has been previously concluded, the effects of ocean warming and acidification are species-specific (Chatzinikolaou et al. 2016, Doney et al. 2020) and differences in susceptibility could be relevant to the species respective micro-habitats or different life strategies (Chatzinikolaou et al. 2021a).

The investigation of morphological properties of marine invertebrates using micro-computed tomography has previously demonstrated that this advanced 3D imaging and analytical approach offers an additional insight into the internal micro-structure of organisms by enabling quantification of specific architectural parameters of the full intact specimen (Chatzinikolaou et al. 2021a). Micro-CT allows the generation of interactive, quantitative, three-dimensional images at a submicron level of resolution which can be further analysed and reveal ecological responses relevant to the shell efficiency of gastropods, as well as to the functionality of the aquiferous system in sponges.

## Figures and Tables

**Table 1.** Average values (± StDev) of morphological parameters measured for the two invertebrate species, *Chondrilla nucula* and *Hexaplex trunculus*, from the two populations (North and South) from micro-CT 3D analysis. The three treatments were the Control (current conditions), the South Aegean Climate Change (SACC) and the North Aegean Climate Change (NACC) scenarios.

**Species**	**Treatment**	**Origin**	**Density**	**Structure separation (um)**	**Structure thickness (um)**	**Closed porosity (%)**	**Open porosity (%)**	**Total porosity (%)**
*C. nucula*	Control	North	29.98 (±1.08)	15.13 (±1.17)	34.60 (±3.66)	2.69 (±1.95)	12.70 (±6.39)	15.15 (±4.51)
*C. nucula*	Control	South	38.09 (±5.37)	14.96 (±2.99)	33.05 (±1.24)	2.77 (±1.27)	13.08 (±5.54)	15.55 (±4.35)
*C. nucula*	SACC	North	30.47 (±1.71)	19.34 (±6.65)	41.92 (±6.53)	3.04 (±0.54)	10.11 (±4.58)	12.86 (±3.95)
*C. nucula*	SACC	South	37.19 (±6.12)	17.52 (±8.18)	35.99 (±6.47)	2.52 (±1.30)	13.86 (±5.71)	16.09 (±4.52)
*C. nucula*	NACC	North	30.91 (±2.83)	14.14 (±3.45)	38.11 (±6.76)	3.72 (±1.51)	9.10 (±8.22)	12.59 (±6.70)
*C. nucula*	NACC	South	36.52 (±2.52)	12.84 (±1.99)	34.90 (±4.29)	3.89 (±1.18)	6.96 (±3.95)	10.60 (±2.99)
								
**Species**	**Treatment**	**Origin**	**Density**	**Structure thickness (um)**	**Closed porosity (%)**			
*H. trunculus*	Control	North	46.77 (±0.53)	140.85 (±10.99)	2.98 (±0.34)			
*H. trunculus*	Control	South	47.02 (±0.65)	129.27 (±8.82)	2.79 (±0.24)			
*H. trunculus*	SACC	North	47.79 (±0.61)	127.87 (±8.44)	2.87 (±0.39)			
*H. trunculus*	SACC	South	47.45 (±0.63)	126.64 (±9.50)	2.66 (±0.33)			
*H. trunculus*	NACC	North	47.91 (±0.93)	129.99 (±12.85)	2.64 (±0.45)			
*H. trunculus*	NACC	South	47.88 (±1.36)	125.38 (±10.24)	2.62 (±0.61)			
